# Blood Eosinophils Subtypes and Their Survivability in Asthma Patients

**DOI:** 10.3390/cells9051248

**Published:** 2020-05-18

**Authors:** Andrius Januskevicius, Egle Jurkeviciute, Ieva Janulaityte, Virginija Kalinauskaite-Zukauske, Skaidrius Miliauskas, Kestutis Malakauskas

**Affiliations:** 1 Laboratory of Pulmonology, Department of Pulmonology, Lithuanian University of Health Sciences, LT-44307 Kaunas, Lithuania; egle.jurkeviciute@lsmuni.lt (E.J.); ieva.janulaityte@lsmuni.lt (I.J.); kestutis.malakauskas@lsmuni.lt (K.M.); 2 Department of Pulmonology, Lithuanian University of Health Sciences, LT-44307 Kaunas, Lithuania; virginija.kalinauskaite@lsmuni.lt (V.K.-Z.); skaidrius.miliauskas@lsmuni.lt (S.M.)

**Keywords:** eosinophils subtypes, lung-resident eosinophils, inflammatory eosinophils, adhesion, survivability, allergic asthma, severe eosinophilic asthma

## Abstract

Eosinophils subtypes as lung-resident (rEOS) and inflammatory (iEOS) eosinophils are different in surface protein expression, functions, response to IL-5 and localization in lungs. rEOS- and iEOS-like eosinophils are found in blood; thus, we aimed to investigate their quantity and survivability in asthma patients. A total of 40 individuals were included: 10 steroid-free non-severe allergic asthma (AA), and 18 severe non-allergic eosinophilic asthma (SNEA) patients, the control group consisted of 12 healthy non-smoking subjects (HS). A bronchial challenge with *Dermatophagoides pteronysinnus* allergen was performed for AA patients and HS. Blood eosinophils subtyping was completed with magnetic beads’ conjugated antibodies against surface CD62L. Eosinophils adhesion to hTERT airway smooth muscle (ASM) cells was measured by evaluating their peroxidase activity and viability by annexin V and propidium iodide staining. We found that the predominant blood eosinophil subtype in AA patients was iEOS, while rEOS prevailed in SNEA patients (*p* < 0.05). Moreover, rEOS demonstrated higher adhesion intensity compared with iEOS in all investigated groups. Both eosinophils subtypes of SNEA patients had higher survivability over the AA group. However, iEOS survivability from AA and SNEA groups was higher compared with rEOS under standard conditions, when rEOS survivability increased after their incubation with ASM cells. Bronchial allergen challenge abolished the dominance of blood iEOS in AA patients and prolonged only iEOS survivability. Though the challenge did not affect the adhesion of any eosinophils subtypes, the direct dependence of rEOS and iEOS survivability on their interaction with ASM cells was revealed (*p* < 0.05). These findings provide the premise for eosinophils subtype-oriented asthma treatment.

## 1. Introduction

Chronic airway inflammation rich in eosinophils is an important feature seen in asthma. Airway and blood eosinophilia is associated with increased rates of asthma exacerbations and more intense treatment [1,2]. Mature eosinophils are blood circulating cells, which, after the appropriate stimulus, migrate into the target tissues, including the gastrointestinal tract, kidney, liver, or lungs [3] and are related to many different disorders, which are interrelated with severity of blood, tissues, and organs eosinophilia [4]. Eosinophils release a high amount of cytokines, chemokines, growth factors, and lipid mediators that affect pulmonary structural cell activity and disturb lung homeostasis [5].

Historically, eosinophils were described as a critical player in host defense, including parasites, viruses, fungi, or bacteria, giving them a destructive inflammatory cell label [6,7]. However, it became clear that steady-state eosinophils can contribute to the immunoregulation and tissue homeostasis as well [7,8,9]. Recently, the existence of two distinct eosinophils subtypes was revealed—lung-resident eosinophils (rEOS), which maturate independently to interleukin (IL) 5, with the primary function to maintain tissue homeostasis, and inflammatory eosinophils (iEOS), which mature in IL-5-dependent manner and are mainly involved in immune responses [10].

Eosinophils’ effect on the airway remodeling in asthma depends not only on the activity but also by their viable number in the lungs. Blood iEOS infiltrate the airways mainly after the environmental stimulus like allergen and leave the airways with bronchial secretions. However, rEOS reside lung tissue for their entire lifetime regulating local immunity [10]. In mice, the model showed that blood rEOS quantity remains stable, while iEOS number increases after *Dermatophagoides (D.) farinae*-induced airway inflammation [10], indicating a different role of eosinophils subtypes in allergic conditions.

Eosinophils stay viable for up to 24 h in blood [11], while being in the lungs prolongs their viability by up to 72 h [12]. Eosinophils’ survivability-promoting factors are mainly associated with cytokines, released by type 2 T helper cells and type 2 innate lymphoid cells [13,14]. There is evidence that the direct interaction of eosinophils with pulmonary structural cells promotes their survivability [15,16,17], but the precise mechanisms remain unknown. The peribronchial area was usually considered as eosinophils localization in asthma [18]. However, eosinophils subtypes differ by their locus—only iEOS are found peribronchially, while rEOS are localized in the lung parenchyma. Due to different localization and behavior of eosinophils subtypes after migration to asthmatic lungs, rEOS and iEOS could vary in adhesion properties. Adhesion is essential for eosinophils infiltration into the airways and could be recognized as a survivability-promoting factor [15,19].

rEOS- and iEOS-like eosinophils were confirmed in the blood of mice, indicating that the differentiation of both subtypes occurs before their recruitment to the lungs. Thus, experiments with blood eosinophils subtypes could give data about their biological role in disease. Moreover, a blood study could provide additional information about possibilities to prevent the harmful effects of distinct eosinophils subtypes before primary damage of the airways occurs. We hypothesized that the quantity of blood eosinophils subtypes could be different in asthma patients and healthy subjects. Moreover, eosinophils adhesion is not only essential for their migration into the airways but interaction with other cells might be involved in the regulation of their activity and survivability. Thus, we investigated the adhesive properties of blood eosinophils subtypes and their survivability as a potential therapeutic target reducing eosinophils effect to asthma pathophysiology.

## 2. Experimental Section

### 2.1. Ethics Statement

All study participants were informed about the study protocol and gave written consent. For working with human subjects, the study protocol was approved by the Regional Biomedical Research Ethics Committee of the Lithuanian University of Health Sciences (BE-2-13). Trial registration: ClinicalTrials.gov Identifier NCT03388359.

### 2.2. Study Population

We recruited 10 steroid-free allergic asthma (AA) patients, 18 severe non-allergic eosinophilic asthma (SNEA) patients who were using high doses of inhaled steroids, and 12 healthy subjects (HS) as a control group. Patients were from the Department of Pulmonology at the Hospital of the Lithuanian University of Health Sciences Kaunas Clinics. All participants were adults—men and women at age 18–50 years old who were informed about the study protocol and signed written informed consent.

The AA group was formed of newly established, untreated (steroid-free) patients with a non-severe course of the disease, approved with a medical history and symptoms for at least 12 months, with a positive skin prick test to *D. pteronyssinus* allergen and positive bronchial challenge with methacholine.

The SNEA group consisted of the patients with an asthma diagnosis for at least 12 months, and a non-allergic phenotype confirmed by negative allergy history, skin prick tests and no specific allergy symptoms, such as watery runny nose, nasal obstruction, urticaria, rashes, conjunctivitis, without dietary restrictions and any symptoms of digestion. Peripheral blood eosinophil counts were higher than 0.3 × 10^9^/L during the screening visit or higher than ≥0.15 × 10^9^/L if there was a documented eosinophil count higher than 0.3 × 10^9^/L in the 12 months before the screening. A severe course of the disease was approved with at least a 12-month treatment of high doses of inhaled steroids combined with long-acting beta-agonist ± long-acting antimuscarinic agent ± episodic use of oral corticosteroids.

The HS was without allergic (had no symptoms of allergy) and other chronic respiratory diseases, with the negative methacholine test.

**Figure 1:**
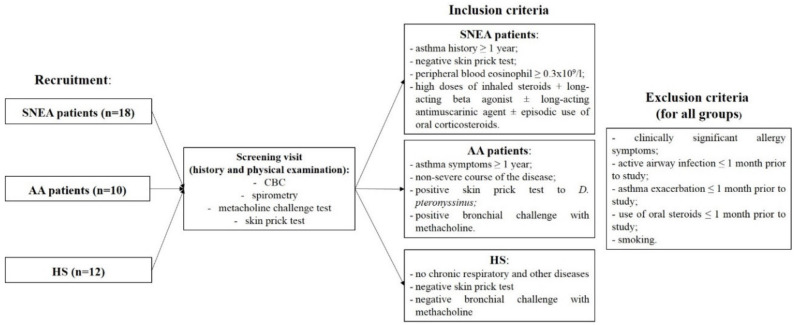
Inclusion and exclusion criteria of the study population. All recruited individuals were newly selected subjects. The inclusion and exclusion were criteria were verified after the screening visit. AA—Allergic asthma; CBC—Complete blood count; HS—Healthy subjects; SNEA—Severe non-allergic eosinophilic asthma.

For all groups, the following exclusion criteria were used: clinically significant allergy symptoms, active airway infection 1 month before the study, exacerbation ≤1 month before study, use of oral steroids ≤ 1 month before study, and smoking. Inclusion and exclusion criteria provided in Figure 1.

### 2.3. Study Design and Experimental Plan

SNEA patients were asked to visit the clinic once, and AA patients and HS patients were asked to visit twice (at baseline and 24 h after bronchial allergen challenge). All study individuals were invited into the study no earlier than 3 days (well-planned preparation of experiments for each study subject was required), but no later than 2 weeks after their inclusion and exclusion criteria were confirmed.

At first visit, for all study subjects, peripheral blood was collected and measured for exhaled fractional exhaled nitric oxide (FeNO). Additionally, AA patients and HS after primary collection of peripheral blood underwent a bronchial challenge with *D. pteronyssinus* allergen.

Initially, the peripheral blood was used for the isolation of granulocytes. Isolated granulocytes were counted and assessed their viability to perform the first quality control of isolation procedures (at least 98% of granulocytes viability and total cells count of >4 × 10^7^). Compliant samples were used for eosinophils enrichment. The second quality control of isolation procedures was performed to isolated eosinophils—eosinophils were counted, evaluated their viability, and assessed purity control by flow cytometer (forward and side light scattering). Passed samples (>1.5 × 10^6^/20 mL blood), viability (>98%), and purity (>96%) were used for eosinophils subtyping. Collected iEOS and rEOS samples were used for third quality control of isolation procedures (>0.5 × 10^6^ cells), viability (>97%).

**Figure 2:**
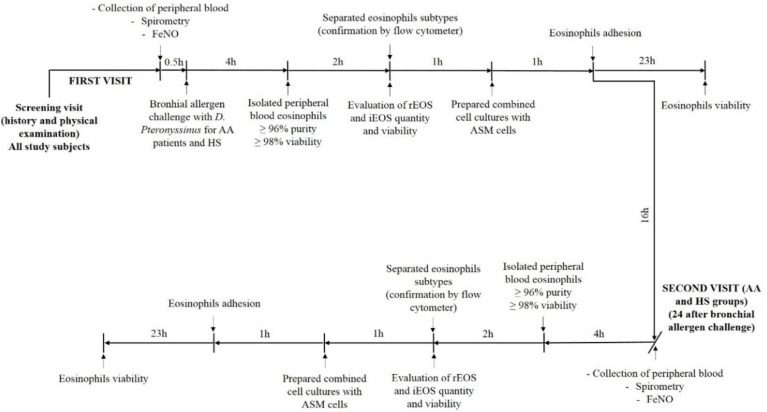
Experimental study design. AA—Allergic asthma; ASM—Airway smooth muscle; FeNO—Fractional exhaled nitric oxide; HS—Healthy subjects; iEOS—inflammatory eosinophils; rEOS—lung-resident eosinophils.

After eosinophils, subtyping combined cell cultures with healthy immortalized ASM cells were prepared immediately, and their adhesive properties were tested after 1 h when adhesion-related viability—after 24 h of incubation. The second visit was 24 h after the bronchial allergen challenge for AA and HS subjects, and all procedures were repeated according to baseline. The study design is shown in Figure 2.

### 2.4. Lung Function Testing

Pulmonary function was tested using an ultrasonic spirometer (Ganshorn Medizin Electronic, Niederlauer, Germany). The results of forced expiratory volume in 1 s (FEV_1_), forced vital capacity (FVC), and the FEV_1_/FVC ratio was considered as the highest of three independent measurements. The data were compared with the predicted values according to age, body height, and sex under the standard methodology.

Pressure dosimeter (ProvoX, Ganshorn Medizin Electronic, Niederlauer, Germany) was used for the detection of airway responsiveness using inhaled methacholine. Aerosolized methacholine was inhaled at 2-min intervals, starting with a dose of 0.0101 mg and increasing it by steps up to 0.121, 0.511, and 1.31 mg until the total cumulative dose was achieved, or received the 20% decrease in FEV_1_ from the baseline. The bronchoconstriction effect of each methacholine dose was expressed as a percentage of the decrease in FEV_1_ from the baseline value. The provocative dose of methacholine causing a ≥20% fall in FEV_1_ (PD_20M_) was calculated from the log dose–response curve by THE linear interpolation of two adjacent data points.

### 2.5. Skin Prick Testing

A skin prick test using standardized allergen extracts (Stallergenes, S.A., France) was used for the following allergens: *D. Pteronyssinus*, *D. farinae*, cat and dog dandruff, five mixed grass pollens, birch pollen, mugwort, *Alternaria*, *Aspergillus*, and *Cladosporium* was performed, as all patients were screened for possible allergies. Diluent saline was used as a negative control, histamine hydrochloride (10 mg/mL)—for the positive control. Skin testing was read 15 min after application. The results of the skin prick test were considered positive if the mean wheal diameter was higher than 3 mm.

### 2.6. FeNO Measurement

Fractional exhaled nitric oxide (FeNO) analysis was performed for all study subjects with an on-line method using a single breath exhalation and an electrochemical assay (NIOX VERO, Circassia, UK), according to guidelines [20]. Patients made an inspiration for FeNO-free air via a mouthpiece, immediately followed by full exhalation at a constant rate (50 mL/s) for at least 10 s. The mean of three readings at the plateau phase was used as the representative value for each measurement. Values that were 25 ppb or more were considered elevated values, according to criteria [20].

### 2.7. Bronchial Allergen Challenge Test

Bronchial allergen challenge was performed with inhaled D. Pteronyssinus allergen (DIATER, Spain) via pressure dosimeter (ProvoX, Ganshorn Medizin Electronic, Niederlauer Germany). The starting point for the assessment of the bronchoconstriction effect was 2 min after nebulized saline inhalation. The allergen was inhaled every 10 min, starting with 0.1 histamine equivalent prick (HEP)/mL allergen concentration, increasing it up to 1.0, 10.0, 20.0, 40.0, 60.0 HEP/mL, interrupting the procedure after achieving a 20% decrease in FEV1 from the baseline. The provocative dose of allergen causing a ≥20% fall in FEV1 (PD20A) was calculated from the log dose–response curve by the linear interpolation of two adjacent data points.

### 2.8. Analysis of Peripheral Blood Cells

A complete blood count test was performed on an automated hematology analyzer XE-5000™ (Sysmex, Kobe, Japan).

### 2.9. Isolation of Eosinophils from Peripheral Blood and Eosinophil Subtyping

Peripheral blood (24 mL) was collected to sterile ethylenediaminetetraacetic acid-containing vacutainer tubes (BD Bioscience, San Jose, CA, USA) and diluted with 1× phosphate-buffered saline (PBS) (GIBCO, Paisley, UK) up to 50 mL and mixed well. Density-gradient centrifugation was performed using Ficoll-Paque PLUS (GE Healthcare, Helsinki, Finland) as the whole blood was layered on Ficoll-Paque reagent and centrifuged at 400× *g* force for 30 min at room temperature. The supernatant was removed, and the bottom layer with granulocytes and erythrocytes was collected. To remove the erythrocytes from the cell suspension, we performed the hypotonic lysis of erythrocytes by adding the half volume of sterile deionized water, gently mixing for not more than 10 s and immediately supplementing the mixture with an equal volume of 2× concentrated PBS and centrifuged at 300× *g* force for 10 min. The procedure was repeated until no red blood cells were left. Isolated granulocytes were counter, and the viability test was assessed.

**Figure 3:**
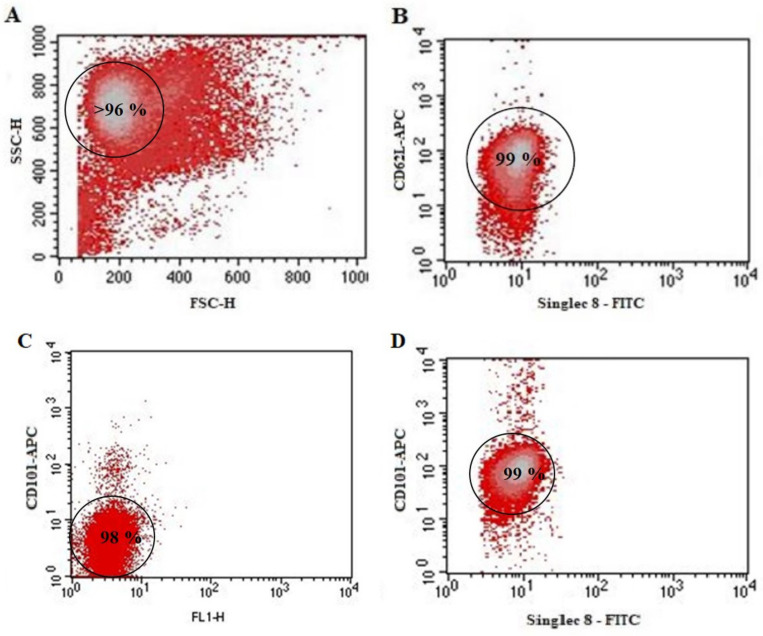
Confirmation of separated eosinophils subtypes by flow cytometry. (**A**) Total enriched eosinophils population; (**B**) separated rEOS population, labeled with CD62L-APC antibody; (**C**) rEOS population labeled with CD101-APC antibody; (**D**) iEOS population labeled with CD101-APC antibody parts; (**B**–**D**) a homogeneous rEOS or iEOS population was used after the final magnetic separation steps. A non-gated enriched total eosinophils population; (**B**–**D**) panels after the gating procedures, excluding cell debris (with SSC/FSC) and non-viable (with propidium iodide) cells. SSC—side scatter; FSC—forward scatter, CD62L—L Selectine, CD101—Immunoglobulin superfamily member 2; Singlec-8—sialic acid-binding Ig-like lectin 8; APC—Allophycocyanin; FITC—Fluorescein isothiocyanate.

Eosinophil enrichment was made with magnetic-activated cell sorting (MACS) by labeling the other granulocytes except for eosinophils with magnetic beads conjugated antibodies (Miltenyi Biotec, Somerville, MA, USA) by the manufacturer’s instruction (the complete procedure is presented in [15]). The manufacturer confirms that eosinophil separation kits do not influence eosinophil viability, and that separation efficiency is more than 96%; additionally, the quality control was made each time with flow cytometer FacsCalibur (BD, Franklin Lakes, NJ, USA) recording the forward and side scattering, as eosinophils distinguish by their granularity (Figure 3A). Moreover, May-Grunwald Giemsa staining and inspection by light microscopy was performed after using new isolation kits as an internal control.

Eosinophils subtyping was performed by using magnetic beads’ conjugated antibodies (Miltenyi Biotec, Somerville, MA, USA) against CD62L, expressed on rEOS surface, but not on iEOS [10]. After eosinophils enrichment procedures, the suspension of cells was centrifuged at 300× *g* force for 10 min at room temperature and resuspended in 80 μL of diluted 1× MACS BSA stock solution (Miltenyi Biotec, Somerville, MA, USA). A total of 20 μL of CD62L-microbeads antibodies were added into the cells’ suspension (per 1 × 10^7^ cells) and were incubated for 15 min at 2–8 °C. Then, cells were washed with an additional 2 mL of MACS buffer, centrifuged at 300× *g* force for 10 min at room temperature, and resuspended in 500 μL of a buffer. The cell suspension was filled on a magnetic column. Unlabeled passed through cells were identified as iEOS. Labeled rEOS were collected by placing a column out of the magnetic field and adding an additional 500 μL of buffer into the column. The manufacturer confirms that positive separation uses up to 10% of selected surface proteins and does not affect eosinophil activity. Isolated eosinophils subtypes were counted, assessed their viability and quantity in peripheral blood. Collected rEOS and iEOS populations were confirmed by labeling with Allophycocyanin (APC)-conjugated antibodies against CD62L and CD101. Excluding non-viable cells and cell debris, 99% of separated rEOS population was positive to their marker CD62L (Figure 3B) and only 2% was positive to iEOS marker CD101 (Figure 3C). Moreover, 99% of the separated viable iEOS population was positive to their marker CD101 (Figure 3D).

### 2.10. Combined Eosinophils and ASM Cells Cultures

Individual combined cell cultures (co-cultures) between blood iEOS or rEOS and healthy human ASM cells, immortalized by stable expression of human telomerase reverse transcriptase, as described in [21], were prepared. ASM cells were grown on plastic dishes in Dulbecco’s modified Eagle’s medium (DMEM) (GIBCO by Life Technologies, UK) supplemented with streptomycin/penicillin (2% *v*/*v*; Pen-Strep, GIBCO by Life Technologies, Paisley, UK), amphotericin B (1% *v*/*v*; GIBCO, Paisley, UK), and fetal bovine serum (10% *v*/*v*; GIBCO by Life Technologies). Cell cultivation was made under the standard conditions—5% CO_2_ in air at 37 °C with medium renewal every 3 days.

Before experiments, the medium was changed to the serum-free growth medium, supplemented with 1% insulin–transferrin–selenium reagent (GIBCO by Life Technologies) and incubated for 24 h to prevent cell division effect of mediators in serum. The same lines of ASM cells were used for whole investigating subjects not older than 10 passages after unfrozen. The confluence in six-well plates—approximately 1.6 × 10^5^ cells in 24-well plates—4 × 10^4^ cells. Co-cultures were made by adding, respectively, 5 × 10^4^ or 1.25 × 10^4^ of eosinophils subtypes. For the visualization of cell growth and co-cultures, we used an inverted microscope (CETI Inverso TC100, Medline Scientific, Chalgrove, UK) with a 10×/22 mm wide-field eyepiece and phase-contrast 10×/0.25 objective and an installed XM10-IR-2 camera (Olympus, Tokyo, Japan).

### 2.11. Eosinophils Adhesion Assay

ASM cells were seeded in 24-well plates and grown under the standard conditions (5% CO_2_ at 37 °C) for 3 days in fetal bovine serum (FBS and antibiotics supplemented medium until confluency was reached. After that, the medium was removed, and wells were washed twice with warm PBS. The medium was changed to the serum-free growth medium, supplemented with 1% insulin–transferrin–selenium reagent and incubated for 24 h to prevent cell division and effect of mediators in serum. rEOS and iEOS adhesion were measured after 1 h of incubation with ASM cells, which is a sufficient period for eosinophils to adhere in co-culture [22]. After incubation, non-adhered eosinophils were removed with medium, and the remaining cells were washed gently with warm PBS. Eosinophil adhesion was determined by measuring residual eosinophil peroxidase (EPO) activity [23]. The data of adhered eosinophils were normalized to measured EPO activity of fixed rEOS and iEOS numbers to avoid possible errors due to not equally expressed EPO in eosinophils. To assay EPO activity, to each well, 116 μL of DMEM medium without phenol red and 116 μL of EPO substrate (1 mM H_2_O_2_, 1 mM *o*-phenylenediamine, and 0.1% Triton X-100 in Tris buffer, pH 8.0) were added. After 30 min of incubation at 37 °C, the reaction was stopped by adding 68 μL of 4 M H_2_SO_4_ to each well. The results were evaluated after reading the absorbance at 490 nm by a microplate reader. The results were expressed as a % of adhered eosinophil number from max added, calculated from a calibration curve. The added eosinophil number was 1.25 × 10^4^.

### 2.12. Eosinophil Viability Assay

The viability of blood rEOS and iEOS was performed by fluorescent staining with annexin V for apoptotic cells and propidium iodide (PI) for necrotic cells and measured with flow cytometer FacsCalibur (BD, Franklin Lakes, NJ, USA). A six-well plate was used for the experiments—three wells were seeded with ASM cells and grown until confluency (1.6 × 10^5^ cells). On the day of experiments, a co-culture with 5 × 10^4^ of isolated rEOS or iEOS was prepared in the serum-free growth medium; additionally, control eosinophil subtypes were seeded in wells with serum-free growth medium without ASM cells. After 24 h of co-culturing, eosinophils were collected into 2 mL centrifuge tubes (Corning Inc., New York, NY, USA), together with eosinophils incubated alone at the same conditions and centrifuged at 300× *g* for 10 min. Additionally, periodical control procedures were performed. Detached ASM cells from co-cultures were used to measure a remaining eosinophil count by flow cytometry (side and forward scattering). No more than 5 % of the added eosinophils count was observed.

For the cell viability assay, we used a fluorescein isothiocyanate (FITC) Annexin V Apoptosis Detection Kit II (BD Bioscience, San Jose, CA, USA) and adapted the method according to the manufacturer’s instructions. Before every experiment, we used additional controls of unstained cells, cells stained with FITC annexin V (no PI), and cells stained with PI (no FITC annexin V).

The data were expressed as viable iEOS and rEOS counts of the total collected from the culture well. The data were normalized with supernatant from ASM cells cultured without eosinophils (for detecting cell debris). Negative control—unaffected serum-free growth medium. Eosinophils and ASM cells significantly differ in size and granularity; therefore, appropriate gating on forward and side scattering excludes any remaining culture heterogeneity. The difference between the debris of the negative control and experimental samples was considered as debris of excluded eosinophils after the final stage of apoptosis and necrosis. These eosinophils cannot be included in the final viability data as to variable among repeats; however, only one experiment was excluded from the final cohort due to a well-visible discrepancy of cell debris.

### 2.13. Statistical Analysis

Statistical analysis was calculated with GraphPad Prism 8 for Windows (ver. 8.01, 2018; GraphPad Software Inc., San Diego, CA, USA). Significant differences between two independent groups were determined using the Mann–Whitney two-sided U-test. The Wilcoxon matched-pairs signed-rank two-sided test was used for dependent groups. Wilcoxon signet-rank test was used to compare the results with hypothetical value. The minimum limit for statistically significant values was *p* < 0.05.

## 3. Results

### 3.1. Characteristics of the Study Subjects

We investigated 40 nonsmoking adults (15 men and 25 women): 10 steroid-free non-severe allergic asthma (AA) patients, 18 severe non-allergic eosinophilic asthma (SNEA) patients with high doses inhaled steroids, and 12 healthy non-smoking control subjects (HS). The main demographic and clinical characteristics of the study population are shown in Table 1. SNEA patients were significantly older, compared with other groups. Moreover, SNEA patients distinguished by a significant deterioration of lung function and the highest blood eosinophils count, compared with AA and HS groups. FeNO was equally increased in both the AA and SNEA groups. The IgE levels were significantly increased in AA and SNEA patients, compared with HS, but the highest level was in the AA group.

The bronchial allergen challenge with *D. pteronysinnus* was performed for all AA patients and eight HS patients (Table 2). A significant increase was observed in the peripheral blood eosinophil count in the AA group following allergen exposure, without changes in IgE and FeNO levels. There were no significant changes in clinical data in the HS group.

### 3.2. Blood rEOS and iEOS Quantity at Baseline and after Bronchial Challenge with D. pteronyssinus

**Figure 4:**
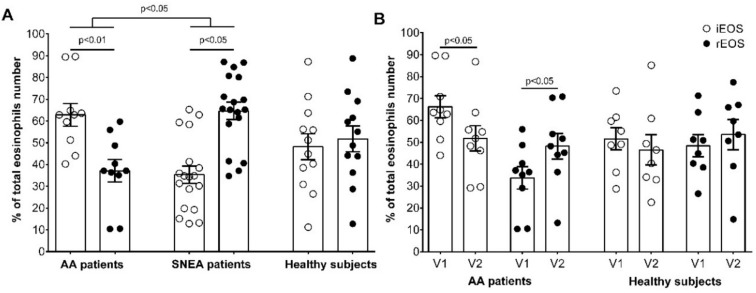
Blood rEOS and iEOS. (**A**) part of rEOS and iEOS in investigated individuals’ peripheral blood. (**B**) part of rEOS and iEOS in investigated individuals’ peripheral blood after bronchial allergen challenge. The results were presented as mean ± S.E.M. AA—allergic asthma; iEOS—inflammatory eosinophils; rEOS—lung-resident eosinophils; SNEA—severe non-allergic eosinophilic asthma; V1—visit 1 (before bronchial allergen challenge); V2—Visit 2 (24 h after bronchial allergen challenge). Eosinophils were counted from: Part (**A**) AA *n* = 10, SNEA *n* = 18, HS *n* = 12; Part (**B**) AA *n* = 9, HS *n* = 8. Statistical analysis: between investigated groups—Mann–Whitney two-sided *U*-test (independent data); within one study group—Wilcoxon matched-pairs signed-rank two-sided test (dependent data), comparing the rEOS and iEOS of each study individual separately.

We isolated the peripheral blood eosinophils and completed their phenotyping with magnetic beads’ conjugated antibodies against CD62L. The predominant eosinophils subtype in AA patients was iEOS—62.8% ± 5.8% vs. 37.2% ± 5.8% of rEOS (*p* < 0.05) of the total isolated eosinophils number. However, the opposite results were found in the SNEA patients group, where the predominant subtype was rEOS—63.8 ± 3.8 vs. 36.2 ± 3.8 of iEOS (*p* < 0.05). There was no significant difference between eosinophils subtypes in the HS group—48.7% ± 5.9% of iEOS vs. 51.3% ± 5.8% of rEOS of total isolated eosinophils number (Figure 4A).

Twenty-four hours after bronchial challenge with *D. pteronyssinus,* the dominance of iEOS in the AA group was repealed, and the proportion of iEOS and rEOS became equal, respectively, 51.8% ± 5.8% of iEOS and 48.2% ± 5.8% of rEOS (Figure 4B). However, the proportion of iEOS and rEOS in the HS group did not change after the allergen challenge.

### 3.3. Adhesion of Blood rEOS and iEOS on ASM Cells

**Figure 5:**
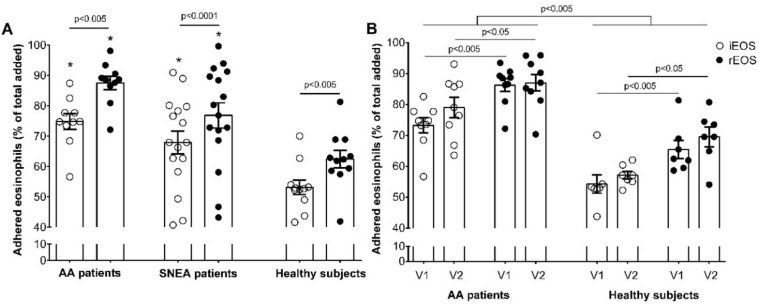
Adhesion intensity of eosinophils subtypes. (**A**) adhesion of blood rEOS and iEOS on ASM cells; (**B**) adhesion of blood rEOS and iEOS on ASM cells after bronchial allergen challenge. The results are presented as the mean ± S.E.M. AA—allergic asthma; iEOS—inflammatory eosinophils; rEOS—lung-resident eosinophils; SNEA—severe non-allergic eosinophilic asthma; V1—visit 1 (before bronchial allergen challenge); V2—Visit 2 (24 h after bronchial allergen challenge). The results from independent experiments of: Part A—AA *n* = 10, SNEA *n* = 16, HS *n* = 11; Part B—AA *n* = 9, HS *n* = 7. * *p* < 0.005 compared with the same eosinophil subtype of HS group. Statistical analysis: between investigated groups—Mann–Whitney two-sided *U*-test (independent data); within one study group—Wilcoxon matched-pairs signed-rank two-sided test (dependent data), comparing the rEOS and iEOS of each study individual separately.

Previously, we demonstrated that asthmatic eosinophils are characterized by increased adhesion to pulmonary structural cells as ASM cells and fibroblasts [15]. In this study, we investigated the adhesive properties of distinct eosinophils subtypes. It was revealed that rEOS are characterized by higher adhesion in combined cell cultures (co-cultures) with ASM cells, compared with iEOS in all investigated groups. After one hour of co-culturing, 87.6% ± 2.2% of rEOS were stable adhered with ASM cells and 74.8% ± 2.6% of iEOS in the AA group (*p* < 0.005); 76.9% ± 4.1% of rEOS and 68.0% ± 4.0% of iEOS in SNEA group (*p* < 0.0001); 62.4% ± 3.2% of rEOS and 53.3% ± 2.6% of iEOS in HS group (*p* < 0.005). There was no significant difference in adhesive properties of eosinophils subtypes between AA and SNEA groups; however, the adhesion of AA and SNEA patients’ eosinophils were increased, compared with the HS group. In the AA group, the number of adhered rEOS was higher by 25.2% ± 2.2% and iEOS by 21.6% ± 2.6%, when in SNEA group, rEOS by 14.5% ± 4.1% and iEOS by 14.7% ± 4.0%, compared with the same eosinophils subtype from the HS group, respectively (*p* < 0.0001) (Figure 5A).

We found that iEOS and rEOS, 24 h after bronchial allergen challenge, did not demonstrate increased adhesive properties, compared with non-activated eosinophils in the AA and HS groups (Figure 5B). However, both eosinophils subtypes of the AA group continued to demonstrate increased adhesion after the challenge, compared with HS—79.0% ± 3.3% vs. 57.1% ± 1.5% of iEOS have stably adhered with ASM cells and 87.0% ± 2.7% vs. 69.5 %± 3.8% of rEOS, respectively (*p* < 0.001).

### 3.4. Blood iEOS and rEOS Viability

**Figure 6:**
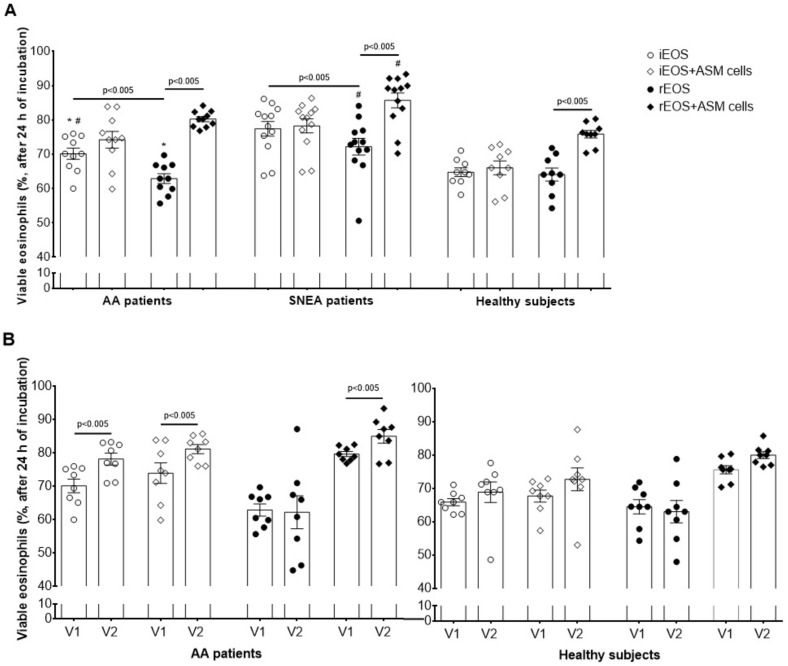
Viability of blood eosinophils subtypes. (**A**) The viability of blood eosinophils subtypes at baseline; (**B**) the viability of blood eosinophils subtypes after bronchial allergen challenge. The results are presented as mean ± S.E.M. AA—allergic asthma; iEOS—inflammatory eosinophils; rEOS—lung-resident eosinophils; SNEA—severe non-allergic eosinophilic asthma; V1—visit 1 (before bronchial allergen challenge); V2—Visit 2 (24 h after bronchial allergen challenge). The results from independent experiments of: Part (**A**) AA *n* = 10, SNEA *n* = 12, HS *n* = 9; Part (**B**) AA *n* = 8, HS *n* = 8. * *p* < 0.05 compared with the same eosinophil subtype of SNEA group, ^#^ *p* < 0.05 compared with the same eosinophil subtype of the HS group. Statistical analysis: between investigated groups—Mann–Whitney two-sided *U*-test (independent data); within one study group—Wilcoxon matched-pairs signed-rank two-sided test (dependent data), comparing the rEOS and iEOS of each study individual separately.

We measured the viability of blood iEOS and rEOS, which were incubated for 24 h in only serum-free growth medium or co-culture with ASM cells. We found that, under the standard conditions (5% CO_2_ at 37 °C, serum-free growth medium), the most viable eosinophils subtype remains iEOS, isolated from SNEA patients; viable iEOS accounted for 77.4% ± 2.1%. It was significantly higher, compared with iEOS, isolated from AA patients—70.1% ± 1.8%, and the HS group—64.8% ± 1.4% (*p* < 0.05). Moreover, iEOS viability was higher, compared with rEOS in AA and SNEA groups, respectively, by 7.2% ± 2.2% and 5.2% ± 2.2% (*p* < 0.05). The results were different if we measured the viability of eosinophils subtypes after 24 h of co-culturing with ASM cells under the standard conditions. We found that co-culturing with ASM cells does not affect the viability of iEOS in all investigated groups; however, it had a significant effect on rEOS viability—in the AA patients group, the rEOS viability increased by 17.4% ± 1.9%, in the SNEA group by 13.5% ± 3.1% and in the HS group by 11.8% ± 2.3% (*p* < 0.01). The adhesion-related effect on rEOS viability was not significantly different between groups (Figure 6A).

Furthermore, we evaluated the iEOS and rEOS viability of AA patients and HS groups 24 h after bronchial allergen challenge. We found a significantly prolonged iEOS viability by 8.0% ± 0.8% in the AA group when they were incubated alone in the serum-free growth medium, and by 7.2% ± 2.3% in co-culture with ASM cells, *p* < 0.05, compared with non-activated iEOS. Moreover, the allergen challenge did not affect the single incubated rEOS viability; however, it prolonged the adhesion-related rEOS viability as viable rEOS number in co-culture with ASM cells increased by 5.3% ± 1.0% (*p* < 0.05), compared with non-activated rEOS (Figure 6B). There was no difference in iEOS and rEOS viability after the allergen challenge in the HS group.

## 4. Discussion

The research of distinct eosinophils subtypes that are differently involved in asthma pathogenesis could give important data for better disease management. In this study, we found that blood iEOS and rEOS–quantity are different among asthma phenotypes, signaling to different pathogenetic pathways involved in the disease development. Moreover, we found out that eosinophils subtypes differ in their adhesive properties and survivability, allowing us to speculate about possible new therapeutic targets against eosinophilia in asthma. Furthermore, we showed that bronchial allergen challenge affects AA patients’ eosinophils subtypes differently—it abolished blood iEOS dominance, increased iEOS survivability, and significantly enhanced adhesion-related survivability of both subtypes.

Eosinophils spent only a short time in the bloodstream, and their viability in the blood is comparatively lower than being in the target tissues [3,11,24]. There are well-described tissue-resident (also called homeostatic) eosinophils from the intestine, adipose tissue, uterus, thymus, mammary gland, and lungs, and all of them were characterized by differently expressed surface proteins [3,25]. However, only rEOS were described as IL-5 independent cells. The data suggest [10,26,27] that basal levels of eosinophils left after absolute IL-5 depletion are a steady-state rEOS population, and anti-IL-5 treatment affected eosinophils are in inflammatory processes involved iEOS (also called type 2 eosinophils). This type of treatment might disturb lung homeostasis as the rEOS population is eliminated as well. We think that a new era of therapies against eosinophilia might be targeted to the imbalanced eosinophils subtypes; however, there are still not enough data about iEOS and rEOS biology.

The current study was based on the fact that eosinophils differentiate and maturate in the bone marrow and are released into the bloodstream in an active form. Therefore, data with blood iEOS and rEOS could give sufficient information about their behavior after infiltration into the asthmatic lungs. There is some evidence that eosinophils can maturate from blood CD34+ progenitor cells [28,29]; however, it represents a very tiny part of total eosinophils count. Moreover, due to the round-shaped nucleus, rEOS might be not fully maturated cells [10]; however, it is an originated cell as the CD34+ cells are excluded in the isolation process as localized between blood plasma and high-density gradient layers with mononuclear cells, while eosinophils are localized in in the bottom layer of granulocytes and erythrocytes. Moreover, treatment of eosinophilia by targeting blood eosinophils quantity or even activity is one step ahead than inhaled medications, as it could completely prevent eosinophil-related damage at the primary stage. Blood eosinophils subtypes were confirmed in mice model [10]; however, there is no information about their existence in human blood, especially in asthma. We separated the rEOS and iEOS to the subpopulations according to the surface expression of cell adhesion molecule CD62L, which are highly expressed only in rEOS, without being detected in iEOS. One of the most important information is the proportion and predominance of eosinophils subtypes in asthma individuals’ blood, as well as in different asthma phenotypes. Interestingly, SNEA and non-severe AA patients were distinct in a quantity of eosinophils subtypes (Figure 4A). SNEA patients, had the predominant blood quantity of rEOS, while non-severe AA patients—iEOS. Moreover, the dominance of iEOS in AA patients was abolished after bronchial challenge with specific allergen, suggesting that a part of the iEOS rapidly infiltrates the airways under the acute AA episode. These important data can avail in modulating the treatment perspectives for severe/non-severe asthma patients.

Asthma phenotypes are different in inflammatory pathways involved in the disease pathogenesis, which determine a different response to environmental triggers, the severity, involved cells, and disease progression [30]; thus, our data suggest that the quantity of blood rEOS and iEOS could be related to a specific type of asthma. Our data of SNEA patients demonstrate that they had predominant blood quantity of rEOS subtype, and this alarms some issues. iEOS can be considered as eosinophils with a negative role, as they have a high expression of several pro-inflammatory genes, while rEOS with positive, as express several genes associated with tissue homeostasis and regulation of immune responses [10]. However, we think that dysregulated proportions of eosinophils subtypes during asthma conditions can lead to the over-expressed homeostatic rEOS functions. Eosinophils constitute a minority of blood white cells in healthy conditions because they leave the bloodstream very rapidly and differentiate from the progenitor cells only in the presence of required signals [7,8]. Our data could not provide the changes in the absolute number of eosinophils subtypes in investigated individuals as their isolation was a multistep process that can influence the final count of eosinophils subtypes populations. However, SNEA patients stand out by the highest blood eosinophil count (Table 1), which confirms that rEOS in SNEA patient blood is the predominant eosinophil subtype in absolute number.

iEOS functions, unlike rEOS, are well described. We can consider that iEOS are historic, highly active pro-inflammatory eosinophils, localized in a peribronchial area. The recruitment of eosinophils to allergen-induced lung tissue is a hallmark of allergic airway inflammation. We found that after bronchial allergen challenge to AA patients the proportion of blood iEOS compared with rEOS significantly decreased (Figure 4B). It could be explained by reduced blood iEOS count due to infiltration into the airways after an acute asthma AA episode. It is known that after allergen challenge to AA patients, eosinophils count increases in the blood (Table 2), and in induced sputum either [31,32]. Sputum eosinophilia might be related to enhanced iEOS infiltration. However, increased eosinophils count in blood in the context of decreased blood iEOS proportion suggests that it could be due to enhanced rEOS population. However, there is still no clear explanation about these mechanisms. Only iEOS infiltration into the airways after allergen challenge was found in a murine model [10]. The possible limitation of our eosinophils subtypes model is, that after enrichment of total blood eosinophil population from granulocytes, rEOS were isolated by positive selection against CD62L, keeping iEOS as CD62L-negative cells. However, not only iEOS do not express cell adhesion molecule CD62L—other tissue-resident eosinophils fall into this category as well [3]. To date, only high expression of CD101 is described as a marker for the iEOS population; however, significantly lower, but still a sufficient amount of CD101 protein rEOS express as well. Thus, significant iEOS separation according to this protein is not possible. For this reason, the iEOS population can be heterogeneous. However, due to the isolated iEOS population heterogeneity, rEOS dominance in SNEA patients may be even more pronounced.

We hypothesized that, for being a resident cell, the adhesion intensity and highly activated adhesion molecules must be essential for rEOS. Previously, we demonstrated that asthmatic eosinophils represent an enhanced adhesion, compared with healthy eosinophils [22,23]. In the current study, we seek to understand which eosinophils subtypes represent higher adhesion intensity and how it alters during the asthma conditions. Our results clearly show that the rEOS subtype distinguished from iEOS by significantly more expressed adhesive properties (Figure 5A) in asthma patients or in the HS group. Adhesion is required for eosinophil infiltration into the airways; thus, iEOS also demonstrated high adhesion properties. Eosinophils migration from the bloodstream into the lungs depends on the activation of eosinophils integrins. Adhesion through integrins leads to eosinophils arrest on endothelium cells, extravasation into the airway wall, and migration through airway tissues into the airway lumen. However, we demonstrated the part of eosinophils subtypes, which could rapidly, within an hour, adhere to other cells or extracellular matrix, probably due to higher active-state integrins composition.

We found that adhesion of both eosinophils subtypes increased during asthma, but without a significant difference according to disease severity. It allows us to assume that eosinophils receive the stimulus from alarmins to migrate into the tissues, that activate their integrins during asthma conditions. However, the firm adhesion is more expressed in rEOS subtypes because the stable adhesion is required for their function, as iEOS adhesive properties can probably be described as rolling and tethering functions required for their migration through tissues. This finding needs to supplement data about the expression of integrins profiles in eosinophil subtypes and their activation states with a reason for understanding which integrins are directly involved in different iEOS and rEOS adhesive properties. Moreover, we investigated the effect of bronchial allergen challenge on iEOS and rEOS adhesion. Interestingly, as both eosinophils subtypes of AA patients after 24 h of activation in vivo maintained their increased adhesion intensity over the HS, it did not affect their adhesion inside the group. Allergen exposure enhanced blood iEOS infiltration into the airways without affecting the rEOS subtype. Moreover, our results suggest that both subtypes are released from the bone marrow into the circulation in a differentiated and primed state without the need for additional activation for their adhesion-dependent infiltration into the airways.

In all pathological cases, adverse-type cells’ impact on disease pathogenesis depends on their quantity and survivability properties. Studies showed that released eosinophils are programmed to die and they need an activation, mainly by eosinophilopoietins in blood [28,33,34]; however, the composition of mediators in lung tissues are different. We found that, without additional survivability-promoting factors, isolated blood iEOS in the AA and SNEA groups, but not the HS group, demonstrated higher survivability compared with rEOS (Figure 6A). This suggests that the activation of iEOS in the blood is sufficient for their function. Moreover, iEOS and rEOS of SNEA patients were found to be more viable compared with the AA group. We presume that a severe course of the disease could be related to prolonged eosinophils viability. The viable rEOS number after 24 h of isolation was found to be around 60%–70% of the total added in vitro, but we hypothesized that viability in vivo must be higher and the additional viability promoting factors are required. rEOS are tissue-residing cells; thus, their viability presumably might be close to the half-life of eosinophil found in lung tissue [3]. However, the determined half-life of eosinophils in lung tissue probably was found on the peribronchial iEOS subtype, as only several studies described the existence of eosinophils in lung parenchyma [35,36,37].

Previously, we demonstrated that the incubation of blood eosinophils with pulmonary structural cells is related to their prolonged survivability [15]. However, as eosinophils subtypes are distinct cells, we need to discern these survivability properties among iEOS and rEOS, which could allow us to better understand their biological properties in asthmatic lungs. We collected the iEOS and rEOS after 24 h of co-culturing with ASM cells and measured the viable cells count. We found that contact with ASM cells was not necessary for iEOS survivability; however, viable rEOS count significantly increased in all investigated groups (Figure 6A). This suggests that rEOS needs an additional trigger to maintain their active functions, which can be adhesion through integrins or a response to ASM cells’ released mediators. We repeated the experiments on iEOS and rEOS survivability after in vivo eosinophils activation by bronchial allergen challenge. Interestingly, in the AA group, it affected the survivability of only iEOS, without and effect to rEOS (Figure 6B), confirming that iEOS are mainly involved in pro-inflammatory responses, while rEOS—not. However, allergen challenge significantly enhanced the adhesion-related survivability rEOS populations (Figure 6B). We presume that allergen-induced asthmatic responses may stimulate rEOS functions, as lung tissue homeostasis is disturbed. Moreover, we found that the survivability of allergen-activated iEOS is also related to the interaction with other cells (Figure 6B). We speculate that the revealed adhesion-related survivability of iEOS during acute asthma may also lead to delay in their release to the bronchial lumen, prolonging their activity in the peribronchial area.

Our study, together with the mentioned possible heterogenous iEOS population, had several more limitations. Other assays applying more detailed controls could be used for additional validation of adhesion-related survivability properties. Moreover, absolute numbers instead of iEOS and rEOS proportions could give more valuable data, especially in the context of bronchial allergen challenge. We evaluated only late phase allergic reaction effects to blood levels and functions of rEOS and iEOS; however, it would be important to evaluate the differences between early- and late-phase reaction-induced changes in eosinophil subtypes’ biology. Furthermore, SNEA patients used high doses of inhaled steroids that might affect the functions of eosinophils; however, there are no precise data about inhaled steroids transition to circulation and direct effect to blood cells. Inhaled steroids reduce the activity of T cells and bronchial epithelial cells, as well as their released pro-inflammatory mediators that can activate the eosinophils [38,39]. Steroid anti-inflammatory effect is found to reduce eosinophils survival [40] and peripheral blood counts; however, severe asthmatics are only limited affected [41] and are described as steroid resistant. The predominant blood rEOS subtype in SNEA patients, as not pro-inflammatory cells, might be less sensitive to steroid treatment. Moreover, the blood eosinophils count, and the adhesion intensity of iEOS was not reduced compared with AA patients, who were not using the steroids, thus allowing us to assume that steroids had an insignificant effect on iEOS as well. Furthermore, it was shown that, in the presence of high levels of IL-5, glucocorticoids promote eosinophils survival in vitro, possibly contributing to steroid resistance [42]. Previously, we demonstrated significantly increased blood serum IL-5 levels in SNEA patients, compared with AA [43]. This suggests that the increase in eosinophil viability, compared with AA patients, might be related to the steroid effect in the presence of IL-5; however, it has not been fully investigated.

There are still only a few studies describing distinct eosinophils subtypes in the lungs or blood. It is the beginning of a new promising research area for better individualized eosinophilic asthma treatment, as well as treatment for other eosinophilic diseases. Eosinophils may affect many other organs, but especially the gastrointestinal tract [4]. It is known that there are specific homeostatic eosinophils population responsible for gastrointestinal homeostasis [44]. It is important to relate the distinct functions and survivability of intestine homeostatic and compare it with the inflammatory eosinophils subtype during diseases such as eosinophilic esophagitis, gastroenteritis and colitis.

In conclusion, our data could be important in providing a better understanding of eosinophil-related asthma pathogenesis and suggesting a treatment approach based on the eosinophils subtype predominancy.

## Figures and Tables

**Table 1:** Demographic and clinical characteristics of the study population.

	AA Patients	SNEA Patients	Healthy Subjects
Number, *n*	10	18	12
Gender, M/F	6/4	4/14	5/7
Age, years	28.8 ± 2.9 ^#^	58.3 ± 2.7 *	34.2 ± 3.0
BMI, kg/m^2^	23.9 ± 1.3	29.0 ± 1.4	25.3 ± 1.1
PD_20M_, geometric mean [range], mg	0.24 [0.12–0.41]	ND	NR
PD_20A_, geometric mean [range], HEP/mL	0.45 [0.08–2.13]	ND	NR ^§^
IgE, IU/mL	349.6 ± 114.5 *^,#^	137.6 ± 6.0 *	25.4 ± 5.9
FEV_1_, L	4.0 ± 0.3 ^#^	1.5 ± 0.17 *	4.1 ± 0.2
FEV_1_, % of predicted	89.6 ± 3.3 ^#^	54.1 ± 5.0 *	105.2 ± 2.1
Blood eosinophil count, ×10^9^/L	0.53 ± 0.08 *^,#^	0.68 ± 0.11 *	0.17 ± 0.02
FeNO, ppb	66.1 ± 11.5 *	42.3 ± 6.2 *	13.2 ± 1.3

AA—allergic asthma; F—female; IgE—immunoglobulin E; M—male; NR—not responded; ND—not done; SNEA—severe non-allergic eosinophilic asthma; F—female; FeNO—fractional exhaled nitric oxide; FEV_1_—forced expiratory volume in 1 s; PD_20M_—the provocation dose of methacholine causing a 20% decrease in FEV_1_; PD_20A_—the provocation dose of allergen causing a 20% decrease in FEV_1_. Data presented as the mean ± standard error of the mean, except PD_20M_ and PD_20A_ provided as the geometric mean (range). * *p* < 0.05 compared with HS group; ^#^ *p* < 0.05 compared with SNEA group; ^§^ bronchial allergen challenge was performed to 8 subjects. Statistical analysis—Mann–Whitney two-sided *U*-test.

**Table 2:** Clinical characteristics of the study population after bronchial challenge with *D. pteronysinnus.*

	AA Patients		Healthy Subjects	
Number, *n*	10		8	
	*Before allergen challenge*	*24 h after allergen challenge*	*Before allergen challenge*	*24 h after allergen challenge*
Blood eosinophil count, ×10^9^/L	0.53 ± 0.08	0.67 ± 0.08 ^&^	0.20 ± 0.04	0.19 ± 0.05
IgE, IU/mL	349.6 ± 114.5	339.9 ± 114.5	31.3 ± 11.2	32.2 ± 12.0
FeNO, ppb	66.1 ± 11.5	83.4 ± 14.2	12.6 ± 1.9	13.0 ± 2.5

AA—allergic asthma; FEV_1_—forced expiratory volume in 1 s; FeNO—fractional exhaled nitric oxide; IgE—immunoglobulin E; the data are presented as the mean ± standard error of the mean; ^&^ *p* < 0.05 compared to data before allergen challenge. Statistical analysis—Wilcoxon matched-pairs signed-rank two-sided test.
